# Iowa Medical Journal

**Published:** 1858

**Authors:** 


### The Iowa Medical Journal.

The readers of the Journal will naturally inquire why this issue has been so long delayed In reply we can only say that the fault has not been with us, but with the publishing office of this city.

With the commencement of the fourth volume a contract was made with the Times office to publish the Iowa Medical Journal for one year. The contract on the part of the editors has been fulfilled promptly in both paying for its publication and furnishing matter at the proper time. On the part of the publishers the issues have not been regular, and this, the 3d No, which was due for September and October, has, owing to the financial difficulties of the office, which resulted in its sale by the Sheriff, prevented the issue at an earlier day. This, we hope, will be a sufficient reason to our subscribers for the delay.

Before closing this subject, however, we wish to say a word or two in a friendly way to our subscribers. If you will take the trouble to refer to the Receipt list contained in each No. of the present volume, you will find the amount of subscription received since the commencement of the third volume with the name of the person having paid attached, amounting in all to less than $250. This is the sum total which has been received to pay the cost of publication of Vol. III and No. 1, 2 and 3, of Vol. IV, making in all nine issues, which cost upon an average $100 each.

Now I appeal to each one of you, our subscribers, and ask if the *Iowa Medical Journal* should not receive more encouraging support? Cannot the Medical profession of Iowa sustain one Medical Journal? A Journal too which has received the hearty commendation of the medical press throughout the Union!

We are ready to admit that stringency in money matters at this particular time make it difficult to command means sufficient to pay all demands, but will those in arrears not make an effort, even at a sacrifice, and forward us our dues.

We are compelled to say to our subscribers that for the present *The Iowa Medical Journal* will only appear quarterly instead of bi-monthly as heretofore. This arrangement will not in any way interfere with the present Volume, only with reference to the time of issue.

With this No. subscribers will have received three numbers of Vol. IV. There yet remains No's. 4, 5 and 6 to complete the Volume, which will be issued quarterly. We hope that on the completion of the present Vol. all our subscribers in arrears may find it convenient to pay up, and that Vol. V. of The Iowa Medical Journal will again visit them Bi-monthly.

There are a great number of medical men in our own and adjoining States, to whom we would be pleased to send our Journal. Will they not become subscribers as well as contributors and aid us in advancing the interests of so honorable a profession.

Any person sending us the names of five subscribers with $10 inclosed, will be entitled to a copy of the Journal for one year.

